# Effectiveness of Laser Therapy in the Management of Recurrent Aphthous Stomatitis: A Systematic Review

**DOI:** 10.1155/2016/9062430

**Published:** 2016-12-18

**Authors:** Min Han, Hui Fang, Quan-Li Li, Ying Cao, Rong Xia, Zhi-Hong Zhang

**Affiliations:** ^1^College & Hospital of Stomatology, Key Lab of Oral Diseases Research of Anhui Province, Anhui Medical University, Hefei 230032, China; ^2^The 2nd Hospital Affiliated to Anhui Medical University, Hefei 230601, China; ^3^The Hospital of Anhui Province, Hefei 230001, China

## Abstract

*Objectives*. Laser therapy is a promising new treatment for patients with recurrent aphthous stomatitis (RAS). However, the clinical effect and security issue of laser therapy remain controversial. This systematic review was conducted to evaluate the clinical effectiveness and security of laser treatment in RAS patients.* Methods*. Five electronic databases were searched (MEDLINE (PubMed), EMBASE, ScienceDirect, the Cochrane Library, and Web of Science) to identify all studies that were about randomized controlled clinical trials, involving the effect of laser therapy in RAS patients.* Conclusion*. Twenty-three studies were retained for full-text analysis after screening the titles and abstracts of potential articles, but only 10 studies satisfied the inclusion criteria after the full texts were reviewed. The included studies reported a comparison of the effectiveness between the laser treatment and placebo laser therapy (or conventional drug therapy) when managing the RAS patients. It can be concluded that laser therapy has the superiority in relieving ulcer pain and shortening healing time when compared with placebo group or medical treatment group. Although laser therapy is a promising effective treatment for RAS, high-quality clinical studies with large sample size must be further performed to confirm the effectiveness of this therapy.

## 1. Introduction

Recurrent aphthous stomatitis (RAS), also known as recurrent aphthous ulcer or recurrent oral ulcer, is the most common recurrent oral mucosal lesions. The prevalence of RAS in the general population is between 2% and 50%; most estimates fall between 5% and 25% [1–3]. RAS clinically manifests as small, round or ovoid, painful, self-healing, and recurrent ulcers with circumscribed margins, erythematous haloes, and yellow or grey floors. RAS may occur during childhood or adolescence, and mucosal lesions may disturb patients' daily activities, such as drinking, eating, and speaking [4]. RAS can be classified into minor (MiRAS), major (MjRAS), and herpetiformis ulcers (HUs) [5]. MiRAS, which comprises more than 80%–90% of RAS cases, presents lesions of less than 1 cm in diameter and heals within 7–14 days without scar formation. MjRAS lesions exceed 1 cm in diameter and heal within 20–30 days with scarring. HUs are characterized by 1–3 mm, multiple, and clustered lesions, which may coalesce into larger ulcers and heal up to 15 days [6]. Although the exact cause of RAS is not completely understood, mucosal lesions are reported to be related to several factors, such as immune system dysfunction, genetic factors, allergic agents, stress, nutrition deficiency, hormonal changes, and infective viruses [7]. Curative therapy is currently unavailable to prevent the recurrence of ulcer [8]. Conventional treatment for RAS involves the use of topical or systemic drugs only to relieve the severity of painful symptoms and prevent secondary infection. Topical agents, such as corticosteroids and other anti-inflammatory agents, including benzydamine, amlexanox, aphtheal, and triclosan, are usually provided for patients with mild symptoms in forms of mouth rinse, adhesive paste, or anesthetic gel [9–12]. However, for those patients with particularly frequent or severe RAS, systemic immunosuppressive treatment (corticosteroids, pentoxifylline, thalidomide, etc.) may be necessary [13–16]. Corticosteroids, antibiotics, and analgesics play the role of mainstay in the treatment of patients with RAS, especially in improving healing of severe RAS [17, 18]. However, long-term or repeated use of these medications should be avoided as fungal infection or drug resistance or even life-threatening complications may be caused [19]. Therefore, many doctors are exploring new treatments for RAS. The history of the investigation and clinical use of laser therapy in medicine goes back to the late 1960s [20]. Recently, laser therapy is clinically accepted in medical fields and practices as part of physical therapy for many diseases such as hair regrowth, infantile hemangioma, incontinent great saphenous vein, and diabetic foot ulcer [21–24]. Besides, it has been applied in dental diseases such as periodontics and peri-implantitis, dentinal hypersensitivity, and dental movement [25–30]. Due to the energy output of laser therapy in a low energy density, it is also named as low-level laser therapy (LLLT) or low-power laser therapy. Thus, the possibility of clinical complications, like thermal effects and soft tissue damage, is reduced. Laser therapy is applied to treat RAS because of its potential beneficial effects, including immediate pain relief, accelerating wound healing, reducing healing period, and being anti-inflammatory [31]. Currently, clinical case reports and randomized controlled clinical trials about several different types of lasers (Nd:YAG laser, Er:YAG laser, InGaAlP laser, GaAlAs laser, etc.) are reported in the use for treatment of RAS.

Although earlier systematic reviews [32, 33] about laser therapy for RAS have been reported, the randomized controlled clinical trials they included were limited. One systematic review [32] identified the randomized controlled clinical trials until 1 June 2014 and only two eligible studies [34, 35] were selected. The other systematic review [33] made an electronic search in databases until 31 December 2013 and four original articles [36–39] were included in this review. Moreover, some important issues of laser therapy on treating RAS, such as clinical security, remain controversial. Therefore, a new systematic review should be conducted. In this work, we would try our best to identify all relevant randomized controlled clinical trials to evaluate the clinical effect and security of laser treatment on RAS.

## 2. Materials and Methods

### 2.1. Search Strategy

The following electronic databases were searched from February 2016 to April 2016: MEDLINE (via PubMed), EMBASE, ScienceDirect, the Cochrane Library, and Web of Science. To identify relevant literature, we searched the following terms “(recurrent oral ulcer OR recurrent oral ulcers OR recurrent aphthous ulcer OR recurrent aphthous ulcers OR recurrent aphthous stomatitis OR recurrent oral stomatitis) AND (laser OR lasers)”. Manual search was also conducted in the following relevant journals published from 2000 to 2016: Lasers in Medical Sciences, Lasers in Surgery and Medicine, Photomedicine and Laser Surgery, Oral Diseases, Photodiagnosis and Photodynamic Therapy, Journal of Oral Rehabilitation, Clinical Oral Investigations, Journal of Dentistry, Journal of Oral Laser Applications, and Journal of Oral Surgery, Oral Medicine, Oral Pathology and Oral Radiology.

### 2.2. Study Inclusion and Exclusion

Inclusion criteria were as follows:

1. Only randomized control trials (RCTs) were considered for the systematic review.
2. Patients with reliable history of RAS, duration of 3 days or less, and presence of one or more painful ulcers were selected for the study.
3. The trial groups received positive laser therapy; the placebo group was conducted with the same laser but with an inactive probe, or the negative control was supplied with some traditional drugs.

Exclusion criteria were as follows:

1. Reviews, case reports, letters, editorials, and conference abstracts were excluded.
2. Patients with oral mucosal ulcers other than RAS, such as ulcerative colitis, Crohn's disease, Behçet's syndrome, or serious anemia were excluded.
3. Studies with insufficient data or without negative group were excluded.
4. Studies published in non-English language were excluded.

### 2.3. Data Extraction and Quality Assessment of Selected Studies

**Figure 1:**
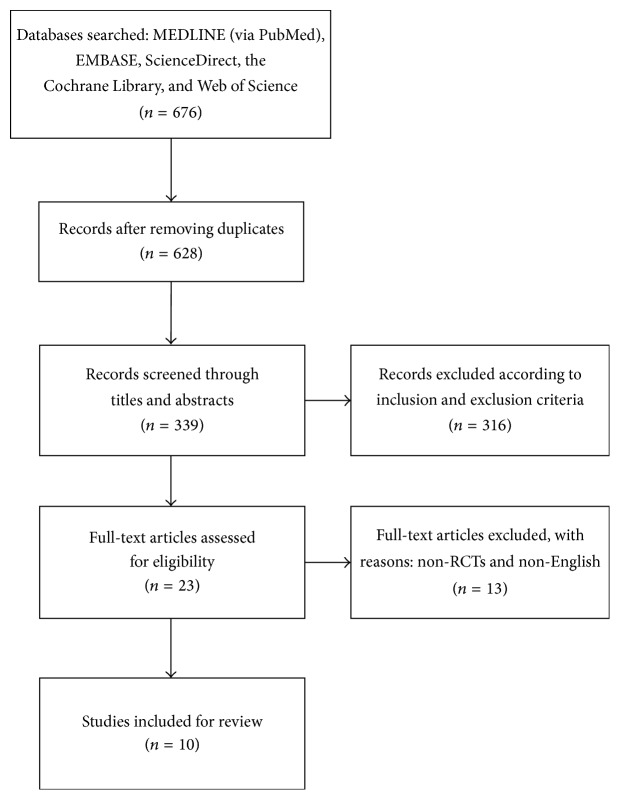
Flow chart showing details of the study selection process.

Figure 1 summarizes the details of the study selection process and the reasons for exclusion. Only studies published in English language and utilized RCT design were selected in the systematic review. Study inclusions and quality assessments were independently conducted by two reviewers between March 2016 and April 2016. Any disagreements were discussed by the two reviewers until consensus was reached. Missing, unclear, or unpublished data were further obtained by emailing the corresponding authors.

Microsoft Excel was used to extract the characteristics of each study included for final analysis. The details of the selected studies included the following: study name (the name of the first author and the year of publication), country of origin, number of ulcer sites, number of patients, age of patients, types of laser, and follow-up period. The outcome evaluation (pain score, healing time, adverse events, etc.) and parameters of various types of lasers were also summarized.

The criteria for the evaluation of RCTs (randomization, allocation concealment, blinding, reporting loss to follow-up/withdrawal, and comparability of baseline) were combined with the Cochrane Handbook for Systematic Reviews of Interventions for the quality assessment of all selected trials (Table 1). The high quality of evidence was estimated when all criteria were met, moderate quality was estimated when one or more criteria were unclear or partly met, and low quality was estimated when one or more criteria were not met [44].

## 3. Results and Discussion

### 3.1. Study Selection

A total of 676 potential relevant titles, abstracts, and articles were found through electronic and manual search. 48 duplicate articles were excluded, and 628 articles were further excluded according to inclusion and exclusion criteria after screening the titles and abstracts. We reviewed the full texts of the remaining 23 articles. Of the 23 articles, twelve full-text articles reported nonrandomized controlled clinical trials and one non-English randomized controlled clinical trial were excluded. Ten studies were finally included in the systematic review [34–43]. The inclusion process is shown in Figure 1.

### 3.2. Study Characteristics

The systematic review included 384 patients. All studies compared the effectiveness of one kind of laser with a negative treatment (an inactive laser therapy or conventional medical therapy). Among these reports, four studies [36–38, 40] made a comparison between the CO_2_ laser treatment and an inactive laser treatment (placebo group). In those studies applied with CO_2_ laser, before irradiation, a layer of transparent, high-water, and nonanesthetic gel was placed on the lesion in patients of the laser and placebo groups. CO_2_ laser is one type of special laser when compared to the conventional low-level laser therapy (Nd:YAG laser, InGaAlP laser, GaAlAs laser, etc.) as its wavelength and energy density are obviously longer and stronger than other lasers. Although it seemed that it is not a low-level laser, the studies of CO_2_ laser therapy in the management of RAS were still included in our systematic review. The importance of the nonanesthetic gel and the reason why we included studies applied with CO_2_ laser therapy will be mentioned in Discussion.

The visual analog scale (VAS) scoring system or a numerical rating scale scoring system, as well as healing time, the size of ulcers, the degree of erythema and exudation (range 0–3), erythema dynamics, and epithelization time, were used to evaluate the outcomes in the included trials. The scoring systems of the VAS and numerical rating scale in assessing pain are usually 0–10 cm or 0–100 mm (the ends of the scale are defined as “no pain” and “severe pain”).

### 3.3. Quality Assessment of Selected Studies

10 studies were finally included in the systematic review [34–43]. As shown in Table 2, five trials [34–36, 39, 42] were defined as moderate-quality evidence and the remaining five trials [37, 38, 40, 41, 43] were rated as low-quality evidence. The randomized method was clearly described in one study [35], in which patients were randomly allocated by tossing a coin. Blinding was conducted in most of the included trials, but only one study [40] was double-blinded and one study [43] was reported to be nonblinded. Only one study [40] reported allocation concealment, whereas the allocation concealment in the remaining nine trials was unclear. Three studies [34–36] reported that these trials were without loss to follow-up/withdrawals. The baselines were comparable in six studies [34–38, 40] and the last 4 trials [39, 41–43] were unclear.

### 3.4. Meta-Analysis Results

This systematic review attempted to conduct a meta-analysis by using RevMan 5.3 software, which could summarize the extracted data from included trials. However, owing to the fact that a quite high heterogeneity was found among the included studies, a meta-analysis was considered to be inappropriate. Therefore, we only provided a descriptive assessment of the included data in the systematic review.

### 3.5. Review

#### 3.5.1. Description of Studies

The characteristics of the included studies, the outcome evaluation of the included studies, and the parameters of various types of lasers are shown in Tables 3, 4, and 5, respectively.

*(i) Laser Therapy versus Inactive “Laser” Therapy*

*(1) Healing Time*. Zand et al. [37] evaluated the healing time of a CO_2_ laser (10600 nm). The healing time after treatment in the laser group (4.8 ± 2.4 days) was dramatically shorter than that in the placebo group (7.6 ± 2.5 days), with *p* value of 0.02.

Prasad and Pai [38] also applied a CO_2_ laser (wavelength not reported) with a significant reduction in the mean healing time, 4.08 ± 0.81 days for the laser treatment group and 7.84 ± 0.90 days for the placebo group (*p* < 0.001).

Aggarwal et al. [34] reported that the complete healing time in the AMD laser group (810 nm) was observed to be 3.05 ± 1.10 days and 8.90 ± 2.45 days in the sham placebo group with *p* value of <0.001.

*(2) Changes in Pain Level*. Zand et al. [36] compared the effectiveness of CO_2_ laser (10600 nm) against that of control group. VAS scores system of 10 cm was used for the evaluation of pain level. Immediately after laser treatment, the mean scores for noncontact and contact pain in the laser therapy group decreased significantly (*p* < 0.001). These mean scores of immediate pain relief in the placebo group were not changed. At 4 h, 8 h, 12 h, 24 h, 48 h, 72 h, and 96 h after laser treatment, these differences of both noncontact and contact pain were significant compared to control groups (*p* < 0.001).

Prasad and Pai [38] compared CO_2_ laser treatment (wavelength not reported) with a placebo laser. In the group treated by laser, the pain level (a numerical analog scale of 0–10) was from 8.48 ± 0.71 (baseline) to 0.68 ± 0.63 (immediately) with a significant reduction (*p* < 0.001) when compared to the placebo group: 8.08 ± 0.70 (baseline) to 7.96 ± 0.84 (immediately). At day 1, the difference was also significant compared to control group.

Sattayut et al. [40] reported the change of pain scores (a 100 mm VAS system) of a CO_2_ laser (10605 nm) group compared to the placebo group. At immediately, day 1, day 2, and day 3 after laser treatment, a statistically significant difference of the pain score between the groups was found only on day 3 (*p* < 0.001). The immediate pain reliefs between the groups were not achieved. Besides, the statistically significant differences were also not observed in the daily activity-disturbance scores between the groups (*p* > 0.05). It seems that there were no statistically significant differences in pain relief between the two groups.

Aggarwal et al. [34] reported a statistically significant reduction in pain by using AMD diode laser unit (810 nm) for RAS patients. The laser group showed a statistically significant reduction of VAS scores (a horizontal 10 cm VAS system) in the immediate pain relief as compared to the sham controlled group. At day 1, day 2, and day 3, the differences were also significant compared to control group.

Albrektson et al. [35] compared the results by using GaAlAs (gallium aluminum-arsenic) semiconductor laser treatment (810 nm) with that placebo laser. Pain scores (a horizontal 100 mm VAS system) and patients' subjective experience of eating, drinking, and brushing were registered. The pain scores changed dramatically with statistically significant difference (*p* < 0.0001) in the laser group compared to the placebo group. The difference was also significant when comparing moderate or severe difficulty with eating, drinking, and brushing between the laser and the placebo group (*p* < 0.0001).

*(3) Size of Ulcers*. Aggarwal et al. [34] found that the AMD diode laser unit (810 nm) group showed a statistically significant reduction in lesion sizes at each follow-up time, especially on day 3 (*p* < 0.001).

Sattayut et al. [40] also reported the change of ulcer sizes after CO_2_ laser (10605 nm) therapy. Clinical data were recorded at baseline, day 1, day 2, day 3, day 5, and day 7 after treatment between the laser group and the control group. However, the authors were disappointed to find that there were no significant difference in the size of ulcers between the laser groups and the placebo groups. It seemed that laser group did not accelerate the wound healing.

*(ii) Laser Treatment versus Medical Treatment*. Tezel et al. [41] compared the results by using Nd:YAG laser treatment (1.064 nm) with conventional medication, a topical corticosteroid (0.1% triamcinolone acetonide given three times daily). The pain degree (evaluated by a 10 cm VAS scale) and erythema and exudation level (the sign of healing) were used to assess the treatment effect. Laser treatment group always presented with a significantly greater efficacy in ulcer pain relieving than medical therapy on days 1, 4, and 7 (*p* < 0.05). As for the pretreatment of topical anesthetic gel to the ulcers area in the laser treatment group, the study did not report the immediate pain relieving after the treatment. Although there was no statistically significant difference in erythema level at any time point during the study (*p* < 0.05), laser therapy group had significantly lower exudation level than medical therapy group at the final patient visit at the end of the study (*p* < 0.05).

De Souza et al. [39] evaluated the effectiveness of InGaA1P diode laser treatment (670 nm) with medication treatment (a topical triamcinolone acetonide given four times daily). The results revealed that no significant difference was noted in RAS regression time between the patients treated with corticoid agent and those treated with laser (*p* = 0.4345).

Lalabonova and Daskalov [42] reported a statistically significant reduction in pain level and erythema and epithelization dynamics (the sign of healing) by using SIX Laser TS diode laser system (658 nm) compared to medication treatment (Granofurin and solcoseryl given twice daily) for RAS patients.

Jijin et al. [43] made a comparison between the laser therapy group (AMD lasers, 810 nm) and the medication treatment group (5% amlexanox oral paste given to the ulcers 4 times daily). Both the participants in 5% amlexanox group and AMD laser group showed a significant reduction in their pain scores and ulcer size on the third day and the seventh day compared with the first day. Also, the intergroup difference in pain levels between the laser group and amlexanox group was significant on the third day (*p* = 0.006); however, the difference in pain levels was not significant on the seventh day (*p* = 0.171). In the meantime, the intergroup difference in the reduction of ulcer sizes was not statistically significant both on the third day (*p* = 0.54) and on the seventh day (*p* = 0.78) after treatment.

*(iii) Clinical Complications*. No adverse reactions or clinical complications were reported in all included ten trials.

### 3.6. Discussion

Although many positive findings were reported in animal models, clinical case reports, and randomized controlled clinical trials, the clinical use of LLLT therapy for oral stomatitis remains controversial. This possibly is due to two main reasons: firstly, the mechanisms of the treatment effects are incompletely understood now; and, secondly, the complexity of treatment protocols choosing such as wavelength, frequency, power density, pulse structure, and treatment timing has led to the difficulty in classification and evaluation of the negative studies as well as positive ones. The exact mechanisms of LLLT are not completely understood. There are several theories that explain the mechanisms of LLLT at present. The adenosine triphosphate (ATP) hypothesis [45] believes that the laser light is absorbed by cytochromes in the mitochondria and then converted into energy—ATP. The cell thus enters a photobioactivated state, during which the target photo activating cellular can use the energy to stimulate its membrane or organelles. Besides, the enhancing level of ATP causes hyperpolarization of the neurons and obstruction of pain stimuli, resulting in an obvious decrease in the induction of pain stimuli, leading to a result of symptomatic pain relief. In addition to the ATP hypothesis, other mechanisms such as the singlet oxygen hypothesis, the redox properties alteration hypothesis, and nitrous oxide (NO) hypothesis may be operating in LLLT [46]. Generally, the mechanisms of low-level light therapy should be continually explored in the future.

This systematic review assessed the actual effectiveness of laser treatment on RAS by analyzing 10 RCTs. As you see, despite the application of various types of lasers with different laser parameters, the results indicated that the laser therapy group showed significant changes on healing time, pain level (especially the immediate pain relief), and the size of ulcers at different follow-up times compared with the placebo group (an inactive laser probe). However, when we tried to draw comparisons between the laser groups and the medical treatment groups, things were going to be different. In those four studies, when talking about the effectiveness in pain relieving, Tezel et al. [41] and Lalabonova and Daskalov [42] reported that laser group showed a statistically significant reduction in pain at different follow-up times, compared to traditional medical treatment. However, Jijin et al. [43] stated that the difference in pain levels between the laser group and amlexanox group was significant on the third day but not significant on the seventh day. When converted to the effectiveness in ulcer healing, Tezel et al. [41] and De Souza et al. [39] conducted that no significant difference was noted between the laser group and medical therapy group, though Lalabonova and Daskalov [42] reported that there was a statistically significant reduction in erythema dynamics and epithelization time (the sign of healing) in the laser therapy group. So it was difficult to make sound conclusions if laser group showed a significantly greater efficacy than medical therapy in the management of RAS.

This review also aimed to explore the security of laser treatment. No visible side effects were reported in the trials included. Some factors may be contributed to the absence of side effects: firstly, the characteristic of the low-power density of the laser beams and, secondly, the use of gel in the application of CO_2_ laser. The laser beams in LLLT, with a narrow spectral width (600 nm–1000 nm) and power densities (1 mw–5 W/cm^2^), are so low that the resulting biological effects are not associated with macroscopic thermal effects [47]. Additionally, it should be noted that, in contrast to conventional low-level laser therapy (Nd:YAG laser, Er:YAG laser, InGaAlP laser, and GaAlAs laser), CO_2_ laser has been used as a very useful high-power, thermal laser in surgery for cutting, ablation, and coagulation of the tissues for many years [31]. However, this high-power laser can also be used as a low-power therapeutic laser in RAS. In the studies about the CO_2_ laser treatment in RAS, before laser irradiation, a thick layer of a transparent, nonanesthetic gel with high-water content is placed on the lesion to reduce the beam absorption by the tissue. The final laser power output is reduced to 2–5 mW which is in the range of low-power lasers, after passing through the gel [31]. As a result, the conventional high-power laser is reduced to a low-power, nonthermal, and noninvasive laser after passing through the gel. This technique is called nonthermal, nonablative CO_2_ laser therapy (NACLT) [37]. Due to the low-level therapeutic nature of NACLT, studies involving the application of low-power nature CO_2_ laser therapy in RAS were included in our systematic review. Although we have observed no visual side effects after the application of laser phototherapy, it should be noted that we should not let down our vigilance against the complications such as the aggravation of the lesions or the thermal damage (tissue ablation, ulceration, and erythema) to the normal oral mucosa. Additionally, some negative experiments reported that DNA damage and reversible cell damage can be observed directly after the laser irradiation in vitro [48]. The amount of DNA damage and cytotoxicity may be related to duration of the laser irradiation, which is dependent on the power density (mW/cm^2^) of each laser.

The results showed a relatively high statistical heterogeneity, maybe owing to various laser types (Nd:YAG laser, Er:YAG laser, CO_2_ laser, etc.) and laser treatment protocols, such as different wave length, output and energy density, frequency, and follow-up period. Furthermore, several limitations must be addressed in this systematic review. Firstly, although most of the included studies provided evidence that laser therapy may help in pain relief and promote wound healing, no report was conducted regarding the difference in recurrence rates after positive and placebo treatments. Secondly, most trials did not report their randomization process and whether treatment allocations were conducted. Nevertheless, treatment allocations may be recognized based on the materials and devices used. Lastly, cost analysis was not performed in this review because no study reported the price of laser therapy.

## 4. Conclusions

In conclusions, although most of studies included in this review reported that laser treatment could significantly alleviate pain (especially the immediate pain relief) and facilitate healing compared with placebo “laser,” it was difficult to make sound conclusions if laser group showed a significantly greater efficacy than medical therapy in the management of RAS. Furthermore, no adverse reactions or clinical complications after the application of laser phototherapy were reported in all included trials. Of course, the evidence of the retrieved studies is weak. Therefore, rigorously designed, long-term, randomized, controlled, and large sample-sized clinical trials must be conducted to confirm the effectiveness of laser on RAS therapy.

## Figures and Tables

**Table 1:** Grade scale for quality assessment of randomized controlled trials.

Criterion		Grade	
	A	B	C
Randomization	Adequate	Unclear (reported randomization but method not described)	Inadequate (quasirandom method of allocation, such as alternation, date of birth, case record number)
Allocation concealment	Adequate	Unclear (not mentioned)	Clearly inadequate concealment/not used
Blinding	Adequate (double-blind or blinding the outcomes evaluators)	Partly blinded/unclear (single-blind or not mentioned)	Not used (“open-label” or “unmask”)
Loss to follow-up/withdrawal	Adequate reporting (including numbers and causes)	Partly reported	Not reported
Comparability of baseline	Comparable (at least including age)	Unclear	Not comparable

**Table 2:** Quality of the included trials.

Criterion	Study									
	Zand et al., 2009 [36]	Zand et al., 2012 [37]	Prasad and Pai, 2013 [38]	Sattayut et al., 2013 [40]	Aggarwal et al., 2014 [34]	Albrektson et al., 2014 [35]	Tezel et al., 2009 [41]	De Souza et al., 2010 [39]	Lalabonova and Daskalov, 2014 [42]	Jijin et al., 2016 [43]
Randomization	B	B	B	B	B	A	B	B	B	B
Allocation concealment	B	B	B	A	B	B	B	B	B	B
Blinding	B	B	B	A	B	B	C	B	B	C
Loss to follow-up/withdrawal	A	C	C	C	B	A	A	A	B	C
Comparability of baseline	A	A	A	A	A	A	A	B	B	B

Quality assessment	Moderate quality	Low quality	Low quality	Low quality	Moderate quality	Moderate quality	Low quality	Moderate quality	Moderate quality	Low quality

**Table 3:** Details of the included studies.

Study	Zand et al., 2009 [36]	Zand et al., 2012 [37]	Prasad and Pai, 2013 [38]	Sattayut et al., 2013 [40]	Aggarwal et al., 2014 [34]	Albrektson et al., 2014 [35]	Tezel et al., 2009 [41]	De Souza et al., 2010 [39]	Lalabonova and Daskalov, 2014 [42]	Jijin et al., 2016 [43]
Country	Iran	Iran	India	Thailand	India	Sweden	Turkey	Brazil	Bulgaria	India
Number of ulcers (test group/control group)	15/15	10/10	25/25	7/7	30/30	20/20	10/10	15/5	90/90	25/25
Number of patients (male/female)	15 (2/13)	10 (1/9)	25	14	30 (18/12)	20	20 (7/13)	20	180 (31/149)	50 (29/21)
Mean ages or age range	37.9 ± 10.9 (24–56)	35.6	(18–40)	(18–39)	Not clear	25	32 ± 7.7	(1–70)	43.01 ± 1.25	(15–55)
Laser group	Nonanesthetic gel + CO_2_ laser	Nonanesthetic gel + CO_2_ laser	Nonanesthetic gel + CO_2_ laser	Nonanesthetic gel + CO_2_ laser	AMD laser	GaAlAs laser	Anesthetic gel + Nd:YAG laser	InGaA1P diode laser	SIX Laser TS diode laser system	AMD laser
Control group	Nonanesthetic gel + placebo “laser”	Nonanesthetic gel + placebo “laser”	Nonanesthetic gel + placebo “laser”	Nonanesthetic gel + placebo “laser”	Placebo “laser”	Placebo “laser”	Medication (triamcinolone acetonide given three times daily)	Medication (triamcinolone acetonide given four times daily)	Medication (Granofurin and solcoseryl given twice daily)	Medication (5% amlexanox given four times daily)
Follow-up time	Immediately, 4 h, 8 h, 12 h, day 1, day 2, day 3, day 4	Not reported	Immediately, day 1	Immediately, day 1, day 2, day 3, day 5, day 7	Immediately, day 1, day 2, day 3	Immediately, day 1, day 2, day 3	Day 1, day 4, day 7	Day 1 to day 7	Day 1, day 2, day 3, day 5,	Day 1, day 3, day 7

**Table 4:** Details of the effects of different intervention measures for RAS patients in the included studies.

Study	Intervention measures	Pain scoring systems	Outcomes	Side effects
	(Laser group/control group)			
Zand et al., 2009 [36]	CO_2_ laser/placebo “laser”	VAS (10 cm) system	*Healing time:* NR *Changes in pain level:* immediately after treatment, these differences were statistically significant between study groups (*p* < 0.001) Laser group: noncontact pain: 6.2 ± 1.3 (baseline) → 0.07 ± 0.3 (immediately); contact pain: 8.4 ± 1.3 (baseline) → 0.7 ± 0.8 (immediately) Placebo group: not changed At 4 h, 8 h, 12 h, 24 h, 48 h, 72 h, and 96 h after laser treatment, these differences of both noncontact and contact pain were significant compared to control group (*p* < 0.001) *Size of ulcers:* NR	No

Zand et al., 2012 [37]	CO_2_ laser/placebo “laser”	NR	*Healing time:* 4.8 ± 2.4 days in the laser group and 7.6 ± 2.5 days in the placebo group (*p* = 0.02) *Changes in pain level:* NR *Size of ulcers:* NR	No

Prasad and Pai, 2013 [38]	CO_2_ laser/placebo “laser”	A numerical rating scale of 0–10	*Healing time:* 4.08 ± 0.81 days in the laser group and 7.84 ± 0.90 days in the placebo group (*p* < 0.001) *Changes in pain level:* Immediately after treatment, these differences were statistically significant between study groups (*p* < 0.001). Laser group: 8.48 ± 0.71 (baseline) → 0.68 ± 0.63 (immediately); Placebo group: 8.08 ± 0.70 (baseline) → 7.96 ± 0.84 (immediately) At day 1, laser group also showed a significant reduction in pain compared to placebo group (*p* < 0.001) *Size of ulcers:* NR	No

Sattayut et al., 2013 [40]	CO_2_ laser/placebo “laser”	VAS (100 mm) system	*Healing time:* NR *Changes in pain level:* Although the pain scores after treatment and daily activity-disturbance scores of the laser group were lower than the placebo group in every point of assessment, a statistically significant difference of the pain score between the groups was found only on day 3 (*p* < 0.001). The immediate pain reliefs between the groups were not achieved. Pain scores after treatment (Laser group; Placebo group): 40.99 (baseline 1) → 42.67 (baseline 2) → 25.39 (immediately) → 38.92 (day 1) → 33.25 (day 2) → 21.45 (day 3) → 3.03 (day 5) → 0 (day 7); 45.17 (baseline 1) → 56.85 (baseline 2) → 32.98 (immediately) → 42.43 (day 1) → 34.98 (day 2) → 33.22 (day 3) → 7.36 (day 5) → 0.44 (day 7) Daily activity-disturbance scores (Laser group; Placebo group): 39.38 (baseline 1) → 43.54 (baseline 2) → 24.25 (immediately) → 34.31 (day 1) → 28.21 (day 2) → 17.98 (day 3) → 0 (day 5) → 0 (day 7); 52.40 (baseline 1) → 65.65 (baseline 2) → 39.70 (immediately) → 52.61 (day 1) → 40.12 (day 2) → 39.82 (day 3) → 7.22 (day 5) → 0 (day 7) *Size of ulcers (mm* ^*2*^ *):* There was no statistically significant difference in the size of ulcers between the laser groups and the placebo groups. Laser group: 4.25 (baseline 1) → 4.00 (baseline 2) → 4.75 (day 1) → 5.25 (day 2) → 7.00 (day 3) → 4.50 (day 5) → 2.00 (day 7); Placebo group: 3.00 (baseline 1) → 4.75 (baseline 2) → 6.25 (day 1) → 6.00 (day 2) → 4.50 (day 3) → 2.50 (day 5) → 1.00 (day 7)	No

Aggarwal et al., 2014 [34]	AMD laser/placebo “laser”	VAS (10 cm) system	*Healing time:* 3.05 ± 1.10 days in the laser group and 8.90 ± 2.45 days in the placebo group (*p* < 0.001) *Changes in pain level:* The laser group showed a statistically significant *reduction in pain scores from baseline values* as compared to the sham controlled group at immediately, day 1, day 2, day 3 after laser treatment. Laser group: 4.79 ± 0.86 (immediately); 4.58 ± 1.2 (day 1); 5.41 ± 2.04 (day 2); 4.72 ± 1.22 (day 3) Placebo group: 0.13 ± 0.35 (immediately); 0.17 ± 0.38 (day 1); 0.48 ± 1.57 (day 2); 0.79 ± 0.62 (day 3) *Size of ulcers (mm):* The laser group showed a statistically significant *reduction in lesion size from baseline values* as compared to the controlled group at immediately, day 1, day 2, day 3 after laser treatment. Laser group: no change (immediately); 0.65 ± 0.6 (day 1); 1.79 ± 0.94 (day 2); 3.17 ± 1.03 (day 3) Placebo group: no change (immediately); 0.10 ± 0.3 (day 1); 0.17 ± 0.38 (day 2); 0.48 ± 0.57 (day 3)	No

Albrektson et al., 2014 [35]	GaAlAs laser/placebo “laser”	VAS (100 mm) system	*Healing time:* NR *Changes in pain level:* the laser group showed a statistically significant reduction in pain scores as compared to the placebo group (*p* < 0.0001) Laser group: 81.7 (baseline) → 56.2 (day 1) → 31.5 (day 2); Placebo group: 84.7 (baseline) → 80.7 (day 1) → 76.1 (day 2) The difference was also significant when comparing the percentage for participants who reported moderate or severe difficulty with daily activities between the laser and the placebo group (*p* < 0.0001) *Size of ulcers:* NR	No

Tezel et al., 2009 [41]	Nd:YAG laser/medication (triamcinolone acetonide)	VAS (10 cm) system	*Healing time:* NR *Changes in pain level:* laser treatment always presented with a significantly greater efficacy in ulcer pain relieving than medical therapy on days 1, 4, and 7 (*p* < 0.05). Laser group: 7.87 ± 0.78 (before treatment) → 1.34 ± 0.76 (day 1) → 0.18 ± 0.23 (day 4) → 0 (day 7) Medication group: 7.72 ± 0.67 (before treatment) → 6.19 ± 0.76 (day 1) → 3.71 ± 0.69 (day 4) → 0.54 ± 0.60 (day 7) The difference was also significant when comparing the pains of daily activity-disturbance such as chewing and speaking between the laser and the medication group (*p* < 0.05) *Change in erythema and exudation level* (signs of healing): there were no statistically significant differences at any time point during the study between groups in erythema. Laser group had a significantly lower exudation (*p* < 0.05) than medication group at the final patient visit at the end of the study Erythema (laser group; medication group): 1.91 ± 0.43 (baseline) → 0.09 ± 0.29 (posttreatment); 2.04 ± 0.48 (baseline) → 0.17 ± 0.39 (posttreatment) Exudation (laser group; medication group): 2.23 ± 0.61 (baseline) → 0.14 ± 0.57 (posttreatment); 2.34 ± 0.77 (baseline) → 0.43 ± 0.51 (posttreatment) *Size of ulcers:* NR	No

De Souza et al., 2010 [39]	InGaA1P diode laser/medication (triamcinolone acetonide)	A numerical rating scale of 0–3	*Healing time:* there was no statistically significant difference in the healing times between the laser group and the medication group (*p* = 0.4345) *Changes in pain level:* NR *Size of ulcers:* NR	No

Lalabonova and Daskalov, 2014 [42]	SIX Laser TS diode laser system/medication (Granofurin and solcoseryl)	A 10-point visual analog scale system	The authors reported the *percentage of patients* in pain levels and erythema and epithelization levels at different time point of assessment (before treatment and on days 1, 2, 3, and 5) *Changes in pain levels:* 0 points (no pain); 1 to 5 points (mild pain); 6 to 10 points (severe pain) Laser group showed a statistically significant reduction in pain levels as compared to medication group in every point of assessment (excepting on day 1, mild pain) with a *p* value < 0.01. *Healing time/change in erythema and epithelization levels (signs of healing)*Erythema levels: erythema, erythema decreases, no erythema; epithelization levels: no epithelization, initial epithelization, epithelization completed At day 3 in laser group, all of the patients were pain-free and had their ulcers successfully treated, while the patients in medication group still felt some pain and their ulcers were failed to be successfully treated even at day 5 Erythema and epithelization processes were evolving through several levels. In nearly every point of assessment (days 1, 4, and 7), laser treatment presented with a significantly greater efficacy in patients than medical therapy (*p* < 0.05) *Size of ulcers:* NR	No

Jijin et al., 2016 [43]	AMD laser/medication (5% amlexanox)	A numerical rating scale of 0–10	*Healing time:* NR *Changes in pain level:* the difference in pain levels between the amlexanox group and the laser group was significant on the third day (*p* = 0.006); however, the difference was not significant on the seventh day (*p* = 0.171) Laser group: 6.80 (day 1) → 5.20 (day 3) → 2.64 (day 7); Medication group: 6.36 (day 1) → 4.16 (day 3) → 1.8 (day 7) *Size of ulcers (mm):* both therapies resulted in a significant reduction in the sizes of ulcers on day 3 and day 7 compared with the first day of treatment. However, the difference was not statistically significant between these two therapies both on day 3 (*p* = 0.54) and on day 7 (*p* = 0.78)	No

NR: not reported.

**Table 5:** Parameters of various types of lasers.

Study	Type of laser	Wavelength	Output power	Energy	Frequency of treatment	Irradiation time	Energy density
Zand et al., 2009 [36]	CO_2_ laser	10600 nm, CW	1 W	NR	NR	5–10 s	NR
Zand et al., 2012 [37]	CO_2_ laser	10600 nm, CW	1 W	NR	NR	5–10 s	NR
Prasad and Pai, 2013 [38]	CO_2_ laser	NR, CW	0.7 W	NR	NR	5–8 s	NR
Sattayut et al., 2013 [40]	CO_2_ laser	10605 nm, CW	2 W	NR	NR	5 s	110.67 J/cm^2^
Aggarwal et al., 2014 [34]	AMD laser	810 nm, CW	0.5 W	NR	NR	45 s; 4 times daily	NR
Albrektson et al., 2014 [35]	GaAlAs laser	809 nm, CW	60 mW	NR	1800 Hz	80 s; once daily	6.3 J/cm^2^
Tezel et al., 2009 [41]	Nd:YAG laser	1064 nm, P	2 W	100 mJ	20 Hz	2-3 min	NR
De Souza et al., 2010 [39]	InGaA1P diode laser	670 nm, P	50 mW	300 mJ	NR	1 min; once daily	3 J/cm^2^
Lalabonova and Daskalov, 2014 [42]	SIX Laser TS diode laser system	658 nm, CW	27 mW	NR	5.8 Hz	1.14 min; once daily	27 J/cm^2^
Jijin et al., 2016 [43]	AMD laser	810 nm	0.1 W	NR	NR	30 s (3 times daily with 2-minute interval)	6 J/cm^2^

P: pulsed wave; NR: not reported; CW: continuous wave.
